# Maladie d'Hallervorden-Spatz chez un adulte jeune

**DOI:** 10.11604/pamj.2014.19.62.5189

**Published:** 2014-09-23

**Authors:** Badreeddine Alami, Siham Tizniti

**Affiliations:** 1 Badreeddine Alami, Service de Radiologie, CHU Hassan II, Fès, Maroc

**Keywords:** Maladie d'Hallervorden-Spatz, symptomatologie, épilepsie myoclonique, Hallervorden-Spatz disease, symptomatology, myoclonic epilepsy

## Image en medicine

La maladie d'Hallervorden-Spatz est une affection autosomique récessive très rare. Elle débute essentiellement entre 5 et 12 ans, mais parfois même dans la première année de la vie. Des cas débutant chez l'adulte jeune ont été rarement rapportés. Sa symptomatologie comporte des signes extra-pyramidaux associant: rigidité, mouvements choréo-athétosiques, dystonie, dysarthrie, détérioration intellectuelle progressive et exceptionnellement une épilepsie myoclonique progressive. Environ la moitié des patients sont porteurs de mutations du gène PANK2, responsable d'une neurodégénerescence associée à la pantothénate kinase ou PKAN. La séméiologie IRM comporte des anomalies de signal bilatérales et symétriques du globuspallidus médial se traduisant sur la séquence pondérée en T2 par un aspect en « oeil de tigre » avec une zone centrale en hypersignal en rapport les lésions neuro-axonales, entourée d'une zone périphérique en hyposignal net en rapport avec les dépôts de fer caractéristiques de la maladie. Cette maladie, pour laquelle il n'existe pas de traitement, conduit à la mort au cours de la deuxième ou troisième décennie de la vie. Nous rapportons le cas d'un jeune homme, âgé de 22 ans, présentant depuis 1 an une dysarthrie associée à une myoclonie et une dystonie des membres inférieurs, chez qui les explorations paracliniques ont été négatives (bilan du cuivre, métabolisme et transport du fer), mais l'IRM cérébrale a objectivé un hypersignal T2 des globi pallidi entouré d'un hyposiganl net. Le diagnostic de la maladie d'Hallervorden-spatz a été retenu. Le patient a été mis sous traitement symptomatique à base de myorelaxants (Baclofène) et de valproate de sodium.

**Figure 1:**
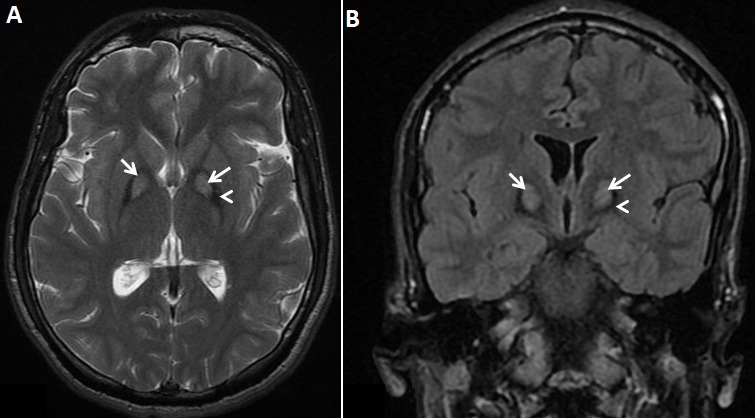
(a) : IRM cérébrale, coupe axiale en séquence T2 objectivant un hypersignal bilatéral et symétrique des globi pallidi (flèche), bordé d'un hyposignal net (tête de flèche) donnant un aspect caractéristique en œil de tigre. (b) : IRM cérébrale, coupe coronale en séquence FLAIR objectivant l'anomalie de signal des globi pallidi se présentant en hypersignal central (flèche) en rapport les lésions neuro-axonales, avec un liseré périphérique en hyposignal (tête de flèche) en rapport avec les dépôts de fer

